# Loss of anchorage primarily induces non-apoptotic cell death in a human mammary epithelial cell line under atypical focal adhesion kinase signaling

**DOI:** 10.1038/cddis.2014.583

**Published:** 2015-01-22

**Authors:** F Ishikawa, K Ushida, K Mori, M Shibanuma

**Affiliations:** 1 Division of Cancer Cell Biology, Department of Molecular Biology, Showa University School of Pharmacy, Tokyo 142-8555, Japan

## Abstract

Anchorage dependence of cellular growth and survival prevents inappropriate cell growth or survival in ectopic environments, and serves as a potential barrier to metastasis of cancer cells. Therefore, obtaining a better understanding of anchorage-dependent responses in normal cells is the first step to understand and impede anchorage independence of growth and survival in cancer cells and finally to eradicate cancer cells during metastasis. Anoikis, a type of apoptosis specifically induced by lack of appropriate cell-extracellular matrix adhesion, has been established as the dominant response of normal epithelial cells to anchorage loss. For example, under detached conditions, the untransformed mammary epithelial cell (MEC) line MCF-10 A, which exhibits myoepithelial characteristics, underwent anoikis dependent on classical ERK signaling. On the other hand, recent studies have revealed a variety of phenotypes resulting in cell death modalities distinct from anoikis, such as autophagy, necrosis, and cornification, in detached epithelial cells. In the present study, we characterized detachment-induced cell death (DICD) in primary human MECs immortalized with hTERT (^Tert^HMECs), which are bipotent progenitor-like cells with a differentiating phenotype to luminal cells. In contrast to MCF-10 A cells, apoptosis was not observed in detached ^Tert^HMECs; instead, non-apoptotic cell death marked by features of entosis, cornification, and necrosis was observed along with downregulation of focal adhesion kinase (FAK) signaling. Cell death was overcome by anchorage-independent activities of FAK but not PI3K/AKT, SRC, and MEK/ERK, suggesting critical roles of atypical FAK signaling pathways in the regulation of non-apoptotic cell death. Further analysis revealed an important role of TRAIL (tumor necrosis factor (TNF)-related apoptosis-inducing ligand) as a mediator of FAK signaling in regulation of entosis and necrosis and a role of p38 MAPK in the induction of necrosis. Overall, the present study highlighted outstanding cell subtype or differentiation stage specificity in cell death phenotypes induced upon anchorage loss in human MECs.

Normal cells undergo cell death and/or growth arrest in the absence of attachment to extracellular matrix (ECM) or upon contact with abnormal or ectopic ECM, which constitutes a physiologically important defense mechanism in multicellular organisms for preventing re-adhesion of detached cells to foreign matrices and their dysplastic growth in inappropriate sites.^1, 2^ On the other hand, the process of cancer metastasis demands that cancer cells circumvent such cell death/growth arrest. This is true even for incipient tumors, where outgrowth and displacement of cells from their original location in a mass result in loss of adequate contact of cells with innate ECM. Cells that disseminate through foreign stroma experience more deviant conditions, and upon reaching the parenchyma of distant organs need to adapt to the non-permissive matrix in the foreign tissue. To survive through this process, cancer cells acquire resistance to cell death/growth arrest induced in the absence of appropriate adhesion to ECM. Therefore, the eradication of cancer cells in ectopic environments requires an understanding of their resistance to anchorage dependence for growth and survival based on responsiveness of their normal counterparts.

Anoikis is a particular type of apoptosis that is induced by inadequate or inappropriate cell–ECM interactions, and is the best-characterized phenotype induced by loss of anchorage in anchorage-dependent epithelial cells.^2, 3^ On the other hand, detachment of cells from ECM has been observed to induce a variety of cell death phenotypes that are distinct from the typical anoikis; these include entosis, autophagy, and squamous transdifferentiation.^4, 5, 6, 7, 8^ The emerging diversity of cell death phenotypes necessitates extension of the study of adhesion-dependent cell death beyond classical anoikis.

A considerable number of studies have suggested that anoikis is the predominant cell death phenotype induced in mammary epithelial cells (MECs) upon anchorage loss;^9, 10, 11, 12, 13^ however, many of these studies employed rodent cells or the human cell line MCF-10 A, which has been characterized as being predominantly myoepithelial or classified into basal B subtype.^14, 15, 16^ Given that the majority of malignant breast cancers exhibit the luminal characteristics, a phenotype based on a normal counterpart or a correspondent luminal subtype of human MECs needs to be defined, particularly given the current limited knowledge in this respect.

In the present study, we characterized anchorage loss-induced cell death in MECs using primary human MECs immortalized with hTERT (^Tert^HMEC).^17, 18^ The established cells are potential stem/progenitors of mammary epithelial cells^18^ and show a partial differentiation toward to the luminal phenotype in the culture system developed by Stampfer *et al* (http://hmec.lbl.gov/mreview.htm). Unlike previous observations based on MCF-10 A cells, the detached ^Tert^HMECs were found to have an apparent defect in the execution of apoptosis and instead, underwent non-apoptotic cell death through simultaneous entosis, cornification, and necrotic processes. The roles of focal adhesion kinase (FAK) and its atypical signaling mediated by TRAIL (tumor necrosis factor (TNF)-related apoptosis-inducing ligand) in this process have been highlighted.

## Results

### Anchorage loss-dependent cell death is induced in ^Tert^HMECs through downregulation of focal adhesion signaling

**Figure 1:**
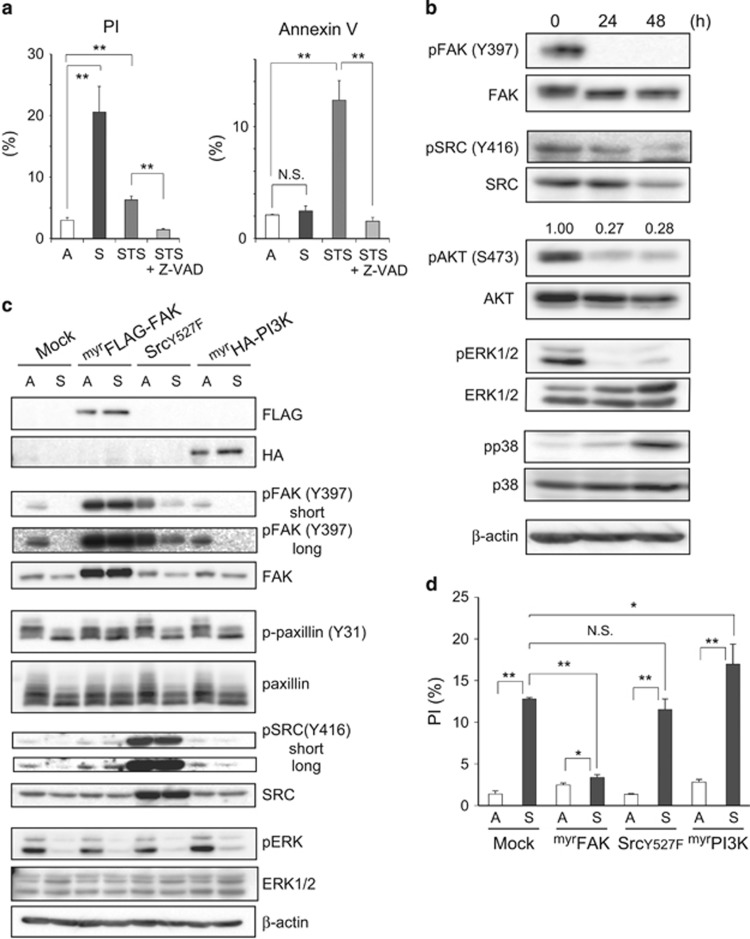
Cell death is induced in suspension cultures of ^Tert^HMECs accompanying downregulation of adhesion signaling. (**a**) ^Tert^HMECs were cultured as monolayers (A) or suspension (S) for 48 h, stained with PI and Annexin V, and subjected to flow cytometry. The percentages of PI- and Annexin V-positive subpopulations are depicted as a graph (means±S.D. from at least three independent experiments). For experimental control, monolayer cultures were treated with 0.5 *μ*M staurosporine (STS) in the presence or absence of 50 *μ*M Z-VAD for 6 h. (**b**) Cells were cultured in suspension for the indicated time periods, and examined by immunoblot analysis using the indicated antibodies. *β*-Actin was used as a loading control. Quantification of band densities was performed using Image J software, and the ratio of the phosphorylated form of AKT to total proteins is shown. (**c** and **d**) Cells were infected with lentivirus constructs expressing control (Mock), FLAG-tagged myrFAK, Src(Y527F), or HA-tagged myrPI3K, and blasticidin selection was performed to obtain cells that stably expressed these proteins. The cells were cultured for 24 h as in (**a**) and examined using immunoblot (**c**). For flow cytometry analysis (**d**), cells were cultured for 48 h, stained with PI and Annexin V, and analyzed as in (**a**). *P<0.05; **P<0.01; NS, not significant

Induction of cell death (hereafter designated as detachment-induced cell death or DICD) was observed in ^Tert^HMECs that were incubated in suspension or in a culture dish coated with the non-adhesive material poly(2-hydroxyethyl methacrylate) or polyHEMA. Approximately 20% of the cells were observed to show positive propidium iodide (PI) staining 48 h after the loss of anchorage (Figure 1a; Supplementary Figure S1a), indicating that a fraction of cells died with concomitant loss of plasma membrane integrity. Supplementation of the suspension culture with exogenous ECM (matrigel) resulted in almost complete abrogation of the increase in PI staining (Supplementary Figure S1b), suggesting that cell death was triggered *per se* by loss of adhesion to ECM and not by an unintentional side effect of the detachment process. In contrast to PI staining, staining with Annexin V resulted in a marginal increase in the fraction of positively stained cells (Figure 1a; Supplementary Figure S1a). Annexin V stains phosphatidylserine exposed in the outer leaflet of plasma membrane, which is an early hallmark of apoptosis. Accordingly, treatment with staurosporine (STS), a typical inducer of apoptosis, increased Annexin V rather than PI-positive cell populations at an early time point (Figure 1a; Supplementary Figure S1a).

The effects of loss of cell-ECM adhesion on intracellular signaling were investigated, with primary focus on FAK and its downstream signaling, which have a central role in cell adhesion-mediated signal transduction.^19, 20^ The survival signals generated upon cell-ECM adhesion are transmitted by FAK and its downstream effectors SRC, phosphatidylinositol-4,5-bisphosphate 3-kinase (PI3K)/AKT, and growth factor receptor-bound protein 2 (GRB2)-MEK/extracellular signal-regulated kinase (ERK) axes.^3, 20, 21^ The initial event in this cascade is a conformational change in FAK that is elicited upon its binding to the cytoplasmic domain of integrin *β*, which leads to autophosphorylation of FAK at Y397 and its concomitant activation. Activated FAK activates SRC, which in turn phosphorylates additional sites in FAK; this leads to the complete activation of FAK, and therefore that of its downstream effectors. However, in detached cells, these signal transduction events are usually downregulated. Figure 1b shows the attenuation of FAK autophosphorylation and activation-dependent phosphorylation of the downstream kinases SRC, AKT, and ERK with a concomitant decrease in expression levels of SRC and AKT in detached ^Tert^HMECs. In marked contrast to other kinases, p38 mitogen-activated protein kinase (MAPK) appeared to be upregulated, as previously reported.^8, 22^

### FAK and its downstream atypical signaling pathway function as a determinant of survival in detached ^Tert^HMECs

To show the causal association between downregulation of FAK signaling and DICD, overriding the signaling downregulation in detached cells was attempted under the assumption that sustained or adhesion-independent activation of survival signaling could counteract DICD. For this purpose, a series of constitutively active forms of kinases associated with the signaling pathway was utilized. Myristoylated FAK (myrFAK), which is known to exhibit adhesion-independent activity,^23^ was first tested. As expected, robust phosphorylation of FAK at Y397 was detected in cells expressing myrFAK (Figure 1c), which was mostly located in the cytoplasm (Supplementary Figure S2a), and phosphorylation became resistant to detachment (Figure 1c). Similar results were obtained for phosphorylation at Y925 and S910 (Supplementary Figure S2b). Consistent with this, paxillin and SRC, which are representative effectors of FAK, were found to be phosphorylated in these cells under detached conditions at levels comparable to an attached cell control (Figure 1c), indicating that FAK transduced signals to the downstream effectors even under conditions of cell detachment. Notably, the cells with such adhesion-independent FAK activity almost completely overcame DICD (Figure 1d; Supplementary Figure S2c), underscoring the important role of FAK signaling in DICD.

Furthermore, constitutively active forms of PI3K (myristoylated PI3K or myrPI3K)^24^ and Src (Y527F mutant or Src(Y527F))^25^ were employed for examining whether DICD could be similarly overcome by these kinases. However, despite expression in cells at detectable levels (Figure 1c), neither myrPI3K nor Src(Y527F) rescued cells from DICD (Figure 1d). Moreover, the expression of myrPI3K failed to have any impact on the phosphorylation of AKT, a downstream effector of PI3K under the condition (unpublished data). Therefore, constitutively active forms of AKT isoforms (myrHA-AKT1, 2, and 3)^26^ were used instead of PI3K. Although myristoylated AKTs were expressed and phosphorylated in cells in an anchorage-independent manner (Supplementary Figure S3a), they failed to reduce DICD (Supplementary Figure S3b), arguing against a major role of the PI3K/AKT pathway in DICD. Similarly, dominant active MEK (MEK2DD) exerted no inhibitory effect on DICD (Supplementary Figure S3c and d). Thus, it was likely that the MEK/ERK pathway was also uncoupled from DICD, which was supported by the observation that myrFAK alleviated DICD with ERK activity remaining downregulated in detached cells (Figures 1c and d). This is in contrast to anoikis in MCF-10A cells, which was dependent on ERK signaling.^12^ Thus, DICD in ^Tert^HMECs was shown to be dependent on FAK activity but not on any of its downstream effectors SRC, PI3K, or GRB2/MEK. Because the ratio of activated *β*1 integrins was rather decreased in detached cells by myrFAK, involvement of inside-out signaling was also unlikely (Supplementary Figure S2d). Although the possibility exists that FAK simultaneously engages multiple downstream pathways for counteracting DICD, with individual downstream pathways being insufficient for cell survival, DICD in ^Tert^HMECs is possibly regulated by atypical and not classical pathways of FAK signaling.

Other signaling molecules, such as ILK, EGFR, ERBB2, p21 protein (Cdc42/Rac)-activated kinase, and Rho family of small G proteins (Rho, Cdc42, Rac), that potentially hae a role in DICD regulation, were also investigated. Among these, EGFR overexpression had a modest effect on DICD, while the overexpression or expression of constitutively active forms of the other molecules, including ERBB2, had essentially no effect (unpublished data).

### Typical apoptosis or anoikis is not detectable in detached ^Tert^HMECs

**Figure 2:**
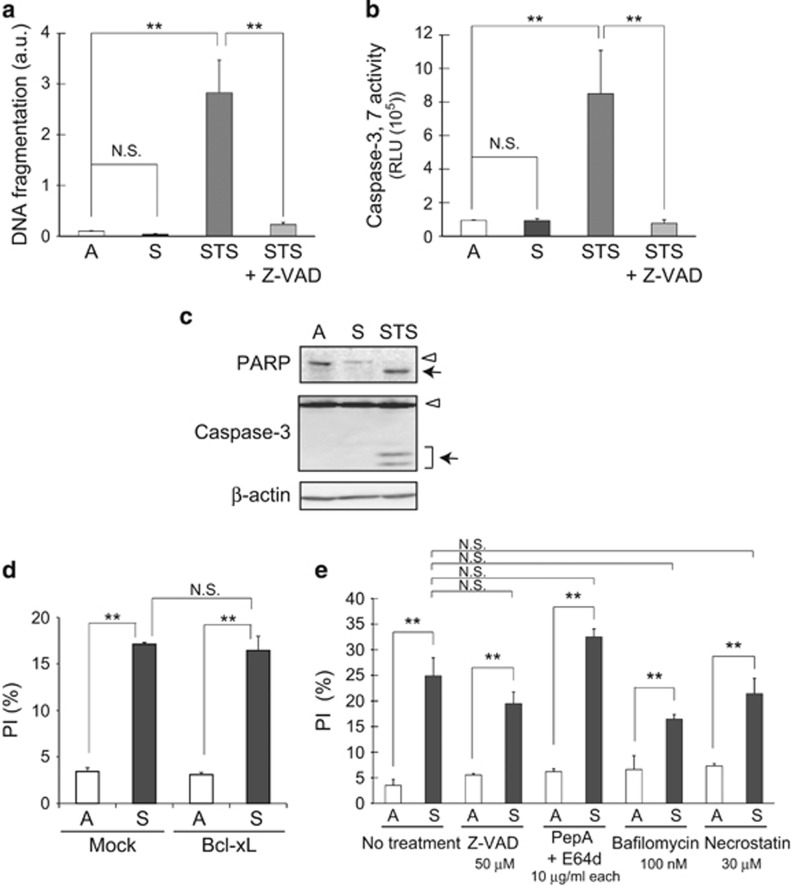
Apoptosis indices are undetectable in detached ^Tert^HMECs. (**a**) DNA fragmentation was quantified in ^Tert^HMECs incubated as monolayers (A) or suspension (S) for 72 h, using the DNA Fragmentation Cell Death ELISA PLUS kit. Values represent means±S.D. from at least three independent experiments, with measurements in triplicate in each experiment. Monolayer cultures treated with 0.5 *μ*M STS in the presence or absence of 50 *μ*M Z-VAD for 24 h were employed as experimental controls. (**b**) Caspase 3/7 activity was assessed in cells cultured for 48 h as in (**a**) using a Caspase-Glo 3/7 assay kit. Representative results from three independent experiments are shown (means±S.D. of triplicate samples). Monolayer cultures treated with 0.5 *μ*M STS in the presence or absence of 50 *μ*M Z-VAD for 17 h were employed as experimental controls. (**c**) Cells cultured for 48 h as in (**a**) were subjected to immunoblot analysis with the indicated antibodies. The arrowhead and arrow indicate intact and cleaved PARP and caspase-3, respectively. Cells treated with STS (0.5 *μ*M) for 17 h were employed as the positive control. (**d**) Cells expressing Bcl-xL were cultured for 48 h as in (**a**), stained with PI and Annexin V, and analyzed by flow cytometry as detailed in Figure 1a. (**e**) Cells cultured in the presence or absence of the indicated inhibitors for 48 h as in (**a**) were stained with PI and Annexin V, and analyzed by flow cytometry as described above. Z-VAD (50 *μ*M), a caspase inhibitor; pepstatin A (PepA) and E64d (10 *μ*g/ml), lysosomal cathepsin inhibitors; bafilomycin (100 nM), a vacuolar H^+^ ATPase (V-ATPase) inhibitor; necrostatin (30 *μ*M), a necroptosis inhibitor. Representative results from three independent experiments are shown (means±S.D. of triplicate samples). **P<0.01; NS, not significant

The lack of dependence of DICD on classical survival signaling and the observation that DICD was not accompanied by an overt increase in Annexin V staining (Figure 1a; Supplementary Figure S1a) suggested that classical apoptosis or anoikis was unlikely to be responsible for DICD in ^Tert^HMECs. In fact, this assumption was corroborated by further experiments. In particular, increase in the typical indices of apoptosis such as DNA fragmentation and activation of caspases, including caspase 3, were not detected in detached ^Tert^HMECs (Figures 2a and b). An active (cleaved) form of caspase 3 or its activity, manifested through cleavage of poly(ADP-ribose) polymerase (PARP), was also not detectable (Figure 2c). Furthermore, the effect of BCL2L1 (Bcl-xL) on DICD was incompatible with the occurrence of apoptosis. As an anti-apoptotic protein of the Bcl-2 family, the expression of BCL2L1 was found to antagonize apoptosis induced by STS (Supplementary Figure S3e and f) but exerted no effect on DICD (Figure 2d). Taken together, these observations supported the conclusion that DICD in ^Tert^HMECs was primarily caspase independent or non-apoptotic, at least under the culture conditions adopted in the present study.

To obtain further information on DICD in ^Tert^HMECs, a pharmacological survey using a set of enzyme inhibitors for a particular type of cell death was conducted. Consistent with the aforementioned results (Figures 2a–d), Z-VAD-fmk (Z-VAD), a broad-spectrum caspase inhibitor,^27^ caused little effect on DICD (Figure 2e). Other inhibitors, such as pepstatin A (PepA), E64d, and bafilomycin for autophagy^7^ and necrostatin for necroptosis,^28^ also exerted only marginal effects (Figure 2e).

### Entosis is observed in detached ^Tert^HMECs

**Figure 3:**
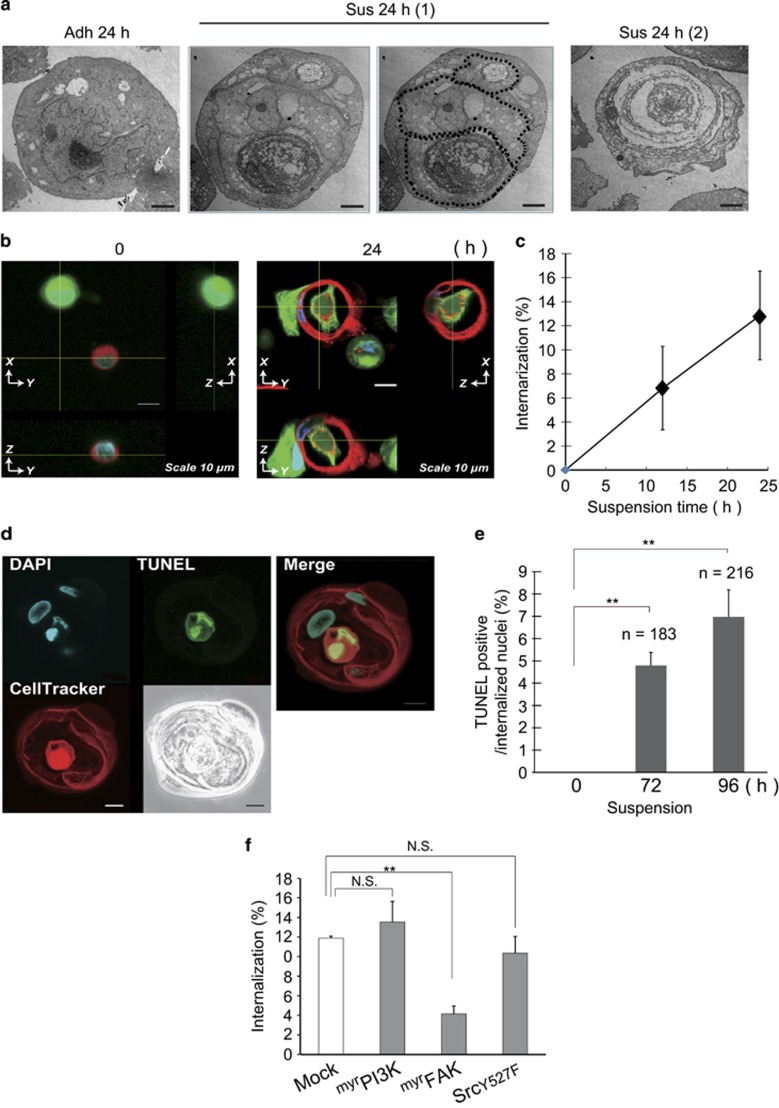
Detached ^Tert^HMECs undergo cell internalization, followed by cell death. (**a**) ^Tert^HMECs cultured for 24 h under adherent (Adh) or non-adherent (Sus) conditions were fixed with 2.5% glutaraldehyde and processed for TEM analysis. Dashed lines outline engulfed cells. Scale bar: 2 *μ*m. (**b** and **c**) Single-cell suspensions of cells labeled with CellTracker Green or Red were mixed in 1 : 1 ratio and plated onto polyHEMA-coated plates. At the indicated time points, cells were stained with Hoechst 33342 (10 *μ*M) and fixed. Confocal images were acquired as z-stacks, processed, and shown as orthogonal views of z-stacks (**b**). Lines on images indicate corresponding points in the orthogonal planes. Three dimensional axes and scale bar (10 *μ*m) are indicated. Percentages of internalization were calculated from 200 cells viewed under a confocal microscope in three independent experiments, and depicted as graph (means±S.D.) (**c**). (**d** and **e**) Cells in suspension culture as in (**b**) were fixed after 96 h (**d**) or 72 h and 96 h (**e**), and TUNEL staining was performed. Confocal images of internalized cells stained with TUNEL (green), CellTracker Red (red), and DAPI (blue) are shown (**d**). Scale bar: 10 *μ*m. Percentages of TUNEL-positive population among the internalized cells were calculated by counting under a confocal microscope, and depicted as a graph (total numbers of cells counted in three independent experiments are shown) (**e**). (**f**) Cells expressing constitutively active forms of various kinases (myrFAK, Src(Y527F), and myrPI3K) were cultured under non-adherent conditions for 48 h as in (**b**). Cell internalization was evaluated as in (**c**), and percentages of internalization were depicted as graphs (means±S.D.). **P<0.01; NS, not significant

With respect to cell death modality responsible for DICD, a morphological study provided clues. As shown in Figure 3a, transmission electron microscopy (TEM) revealed unique ultrastructural changes in the detached population of ^Tert^HMECs; cell internalization within another cell was observed, which closely resembled what has been referred to as cell-in-cell structure,^29^ characterized as the complete inclusion of one cell within another. Such inclusion of cells within other cells was confirmed in detached population of ^Tert^HMECs using red- or green-labeled cell populations which were mixed, incubated in suspension, and visualized by confocal microscopy (Figure 3b). The structure appeared within 12 h, and further incubation resulted in its growth into a large cellular aggregate due to reiterative internalization, which hampered the accurate enumeration of internalization ratio after 24 h (Figure 3c). Nuclear staining with DAPI or TUNEL suggested that cell death was induced in cells inside the structure (Figures 3d and e). Internalized cells have been shown to be degraded by lysosomal activity.^6^ These observations suggest that a cell death modality, which is typified by cell internalization termed entosis, occurs in ^Tert^HMECs upon loss of attachment to ECM. Cell internalization was the only morphological phenotype discernible by TEM in detached ^Tert^HMECs, and this process was suppressed by the expression of myrFAK but not myrPI3K and Src(Y527F), as shown in Figure 3f.

### DICD in ^Tert^HMECs is mediated by at least three types of non-apoptotic cell death modalities

**Figure 4:**
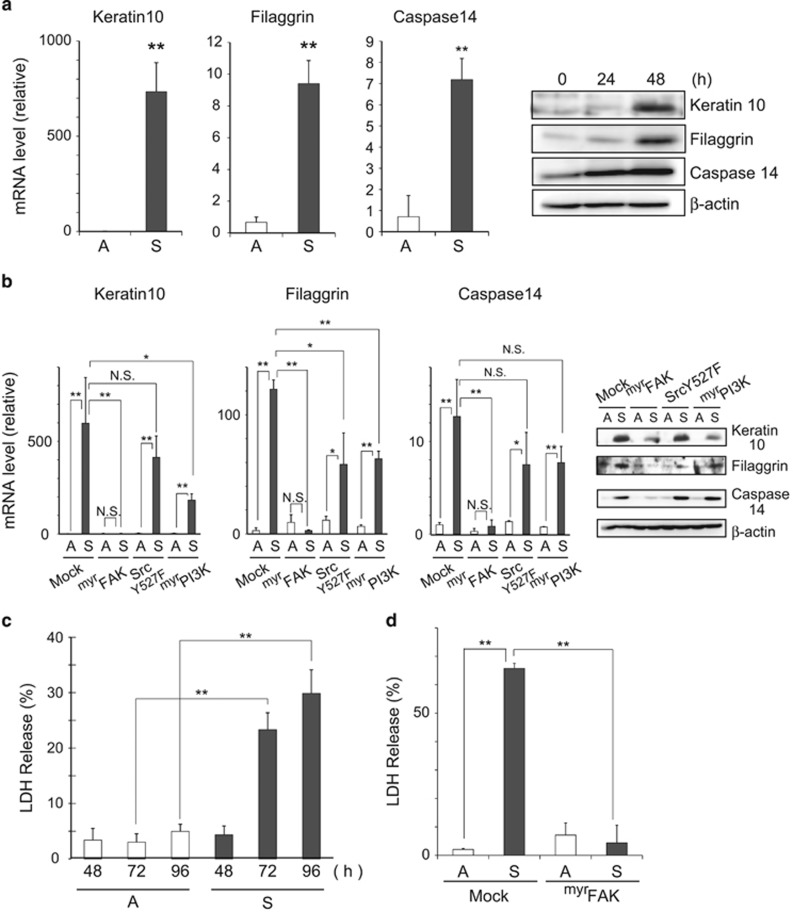
Cornification and necrosis programs are activated in detached ^Tert^HMECs. (**a**) ^Tert^HMECs were cultured as monolayers (A) or suspension (S) for 24 h, following which total RNA was extracted. The levels of various mRNAs were examined by quantitative RT-PCR using the indicated primers (Supplementary Information), and normalized with respect to GAPDH control. Ratio of mRNA levels in suspension (S) cultures to monolayer (A) cultures is shown. Cell lysates from the indicated periods were analyzed by immunoblotting using the indicated antibodies. *β*-Actin was used as a loading control. (**b**) Cells expressing constitutively active forms of various kinases were cultured as in (**a**), and mRNA and protein levels were examined as above. The ratios of mRNA levels with respect to those in monolayer cultures of Mock-infected cells are graphed. (**c**) Cells were cultured as above for the indicated time periods, and LDH activities were measured as described in the Materials and Methods. LDH release (%) represents the ratio of LDH activity in media to total (media and cells) activity. (**d**) Cells expressing myrFAK were cultured as above for 72 h and LDH release (%) was examined as in (**c**). Values represent means±S.D. from at least three independent experiments, with measurements in triplicate in each experiment. *P<0.05; **P<0.01; NS, not significant

Gene expression profiling and biochemical analysis were performed for more precise characterization of DICD. Gene expression profiling suggested that caspase 14, a non-canonical caspase specifically associated with terminal differentiation in keratinocytes,^30^ was upregulated in detached ^Tert^HMECs (unpublished data). Its upregulation was confirmed by quantitative RT-PCR and immunoblotting along with the induction of cornification markers, such as keratin 10 and filaggrin (Figure 4a). This finding is in good agreement with previous observations of epidermis-like cornification in MECs.^8, 31^ Similar to entosis, the expression of these cornification markers was remarkably inhibited by myrFAK expression (Figure 4b). The expression of SrcY527F and myrPI3K also reduced the expression, but their effects were modest and not significant in some cases.

On the other hand, biochemical analysis suggested that DICD assumed features of necrosis, as manifested by the release of lactate dehydrogenase (LDH), which was increased 72 h postincubation under detached conditions (Figure 4c); this increase was also suppressed by expression of myrFAK (Figure 4d). Before LDH release, a decrease in ATP/ADP ratio accompanying the deterioration of mitochondrial membrane potential (ΔΨm) was observed (Supplementary Figure S4a and b). According to a recent report,^4, 32^ MECs suffered metabolic defects under detached conditions, which resulted in necrotic cell death.

Collectively, three simultaneous types of cell death, entosis, cornification, and necrosis, were possibly implicated in DICD of ^Tert^HMECs, and all three types of cell death were mitigated by the expression of myrFAK. In conclusion, DICD in ^Tert^HMECs is mediated by at least three types of non-apoptotic cell death modalities that were presumably primed by downregulation of FAK activity upon loss of anchorage.

### Role of TRAIL in DICD of ^Tert^HMECs

**Figure 5:**
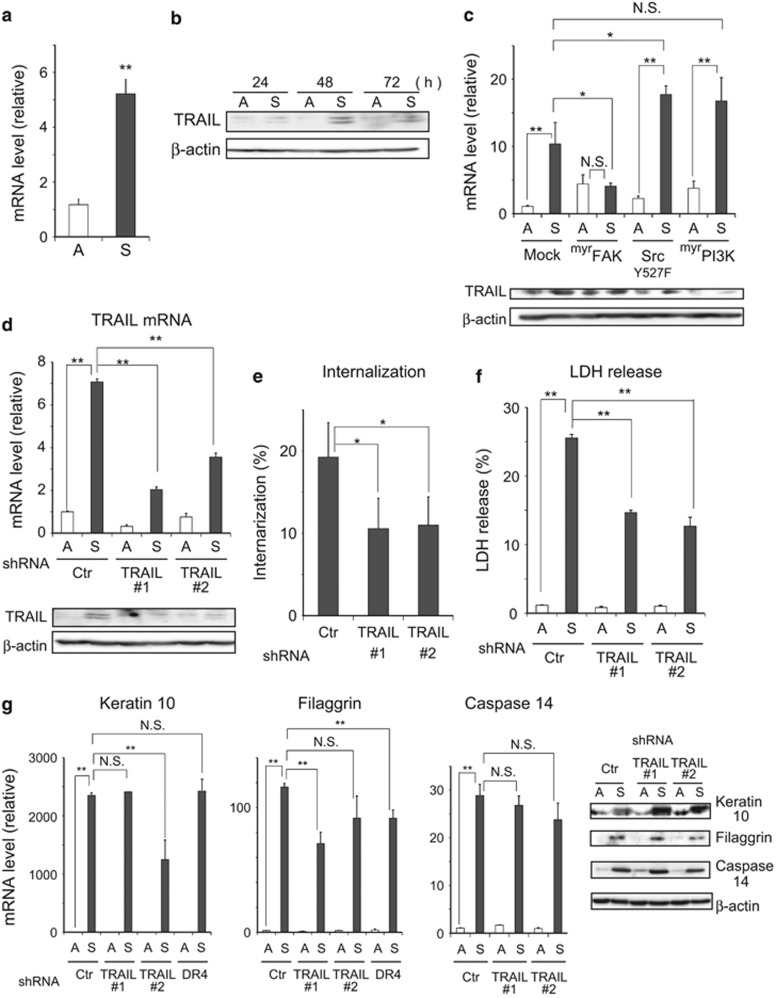
TRAIL upregulation in detached ^Tert^HMECs contributes to cell internalization and necrosis. (**a**) ^Tert^HMECs were cultured as monolayers (A) or suspension (S) for 24 h, and TRAIL mRNA levels were evaluated as in Figure 4a. (**b**) Cells cultured as in (**a**) for the indicated time periods were examined by immunoblot analysis using TRAIL-specific antibody. *β*-Actin was employed as a loading control. (**c**) Cells expressing constitutively active forms of various kinases were cultured as above and TRAIL mRNA and protein levels were examined. The ratio with respect to mRNA levels in monolayer cultures of Mock-infected cells is shown. (**d**) Cells infected with lentiviral expression constructs of shRNAs (Ctr: control, TRAIL # 1, # 2: two shRNAs with unrelated sequences specific for TRAIL (Materials and Methods)) were cultured as in (**a**) and TRAIL mRNA levels were analyzed as above. Cell lysates from 48h cultures were analyzed by immunoblot using TRAIL-specific antibody as mentioned in (**b**). (**e**) shRNA-expressing cells were stained with 10 *μ*M CellTracker Red or Green, mixed, and cultured in suspension for 20 h, followed by counting of internalized cells by confocal microscopy, as mentioned in Figures 3b and c. (**f**) shRNA-expressing cells were cultured as in (**a**) for 72 h, and LDH release (%) was examined as mentioned in Figure 4c. (**g**) shRNA-expressing cells were cultured as in (**a**) for 24 h, and mRNA and protein levels were analyzed as described in Figure 4a. Values represent means±S.D. from at least three independent experiments, with measurements made in triplicate for each experiment. *P<0.05; **P<0.01; NS, not significant

Compared with apoptosis, non-apoptotic cell death and its modalities, regulatory signaling, and mechanisms are poorly understood. In the present study, an important cue was obtained for regulatory signaling in DICD from the results of the aforementioned DNA microarray-based genome-wide screening; TRAIL was found to be upregulated during DICD along with caspase 14. As shown in Figures 5a and b, quantitative reverse transcription (RT)-PCR and immunoblot analyses verified the induction of TRAIL; the induction was detected at the mRNA level as early as 3 h following detachment (Supplementary Figure S5a) and persisted for 72 h (Figure 5b). The expression of myrFAK but not Src(Y527F) or myrPI3K resulted in significantly impaired induction of TRAIL (Figure 5c). shRNA-mediated knockdown of TRAIL expression interfered with cell internalization and LDH release (Figures 5d–f), while the expression of cornification markers was not significantly affected (Figure 5g). DR4 was inferred to function as a receptor for TRAIL (Supplementary Figure S5b–d). These results suggest that in detached ^Tert^HMECs, TRAIL is induced upon downregulation of FAK signaling, and in turn contributes to entosis and necrosis but not cornification mechanisms. Thus, an important role of TRAIL as a mediator of FAK signaling that regulates DICD in ^Tert^HMECs has emerged.

### Role of p38 MAPK in DICD of ^Tert^HMECs

**Figure 6:**
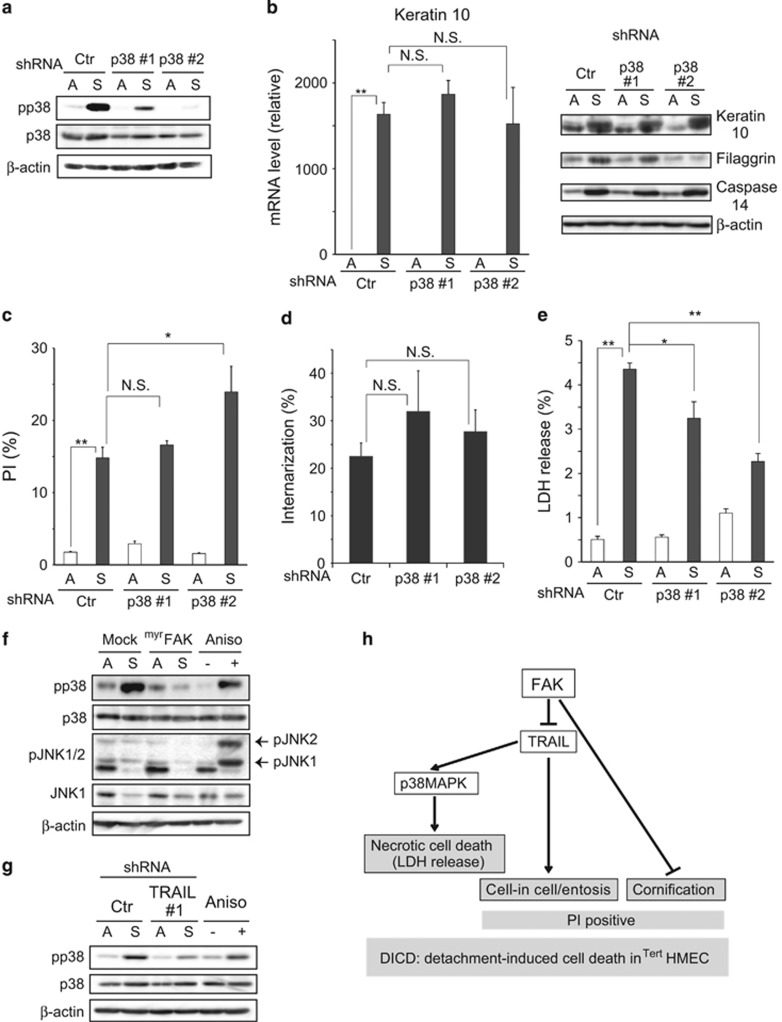
p38 MAPK has a role in inducing necrosis in detached ^Tert^HMECs. (**a**) ^Tert^HMECs expressing shRNAs (Ctr: control, p38 MAPK # 1, # 2: two shRNAs with unrelated sequences specific for p38 MAPK; Materials and Methods) were cultured as monolayers (A) or suspension (S) for 48 h, and examined as described above by immunoblot analysis using the indicated antibodies. *β*-Actin was employed as a loading control. (**b**) Total RNA was extracted from cells cultured for 24 h as in (**a**), and keratin 10 mRNA levels were analyzed as described above. Cell lysates were analyzed by immunoblotting using the indicated antibodies. *β*-Actin was used as a loading control. (**c**) shRNA-expressing cells were cultured as in (**a**), followed by staining with PI and annexin V, and subjected to flow-cytometry analysis, as described in Figure 1a. (**d**) shRNA-expressing cells were stained with 10 *μ*M CellTracker Red or Green, mixed, and cultured for 20 h in suspension. Internalized cells were counted using confocal microscope as described in Figures 3b and c. (**e**) shRNA-expressing cells were cultured as in (**a**) for 72 h, and LDH release (%) was examined as mentioned in Figure 4c. (**f** and **g**) Cells expressing myrFAK (**f**) or shRNA (Ctr: Control, TRAIL # 1) (**g**) were cultured as in (**a**) for 48 h, and examined by immunoblot analysis using the indicated antibodies. Anisomycin (50 *μ*g/ml, 30 min) was used as a positive control for the phosphorylation of p38 MAPK and JNKs. *β*-Actin loading control was used. Values represent means±S.D. from at least three independent experiments, with measurements made in triplicate for each experiment. *P<0.05; **P<0.01; NS, not significant. (**h**) Schematic illustration of FAK-dependent detachment-induced cell death (DICD) in ^Tert^HMECs

The possible involvement of p38 MAPK in the regulation of DICD was investigated. As noted above, p38 MAPK was distinct from other adhesion-related signaling molecules, including JNK, in that it was evidently activated in response to loss of anchorage (Figures 1b and 6f). In addition, a previous study using an inhibitor, SB203580, of p38 MAPK suggested a role for this kinase in activation of cornification program in detached MECs.^8^

In fact, the induction of the cornification markers was similarly inhibited with the inhibitor in detached ^Tert^HMECs in the present study (Supplementary Figure S6). However, the results were different when the activity of the kinase was attenuated with shRNA (Figures 6a and b). The inconsistency between the results of the two approaches is possibly attributed to the limitations of pharmacological inhibition with respect to the specificity of targets. As shown in Figure 6b, shRNAs for p38 MAPK failed to inhibit the expression of the cornification markers, arguing against a role of the kinase in activation of cornification program in detached ^Tert^HMECs. Likewise, the ratio of PI-positive cells and the frequency of cell internalization was not reduced by shRNA expression (Figures 6c and d); only LDH release was suppressed (Figure 6e), suggesting a role for this kinase in necrosis but not entosis and cornification processes. When the association of p38 MAPK activation with FAK and TRAIL signaling was examined, the activation was observed to be significantly impeded by myrFAK expression and shRNA for TRAIL (Figures 6f and g); this observation together with that in Figure 5c suggested that the activation of p38 MAPK was mediated by TRAIL under the downregulation of FAK activity.

## Discussion

### Diversity of DICD in epithelial cells

The diversity of phenotypes assumed by cells upon loss of ECM attachment has been perceived from the seminal study on anoikis, which mentioned different phenotypes in the epithelial cells of gut *versus* ureters.^33^ In the two decades that have elapsed since this study, our knowledge of detachment-responsive phenotypes in cells, particularly in MECs, has been enormously expanded by a series of studies on lumen formation in mammary glands during morphogenesis.^8, 11, 34, 35, 36^ Experiments in mice that were genetically deficient for caspase-dependent cell death mechanisms revealed the requirement of caspase-dependent cell death for efficient luminal clearing but not the eventual formation of a lumen, suggesting that caspase-independent or non-apoptotic cell death programs function as an alternative mechanism for luminal clearing in the absence of apoptosis.^8^ Subsequent studies led to the discovery of autophagy, necrosis, and cornification as such alternatives.^4, 34^

A salient observation of the present study is that immortalized human MECs, bipotent progenitor-like cells with a differentiating phenotype to luminal cells (^Tert^HMECs)^18^ (http://hmec.lbl.gov/mreview.htm), primarily underwent non-apoptotic instead of apoptotic cell death under detached conditions, at least in forced suspension cultures *in vitro*. This mechanism is in striking contrast to MCF-10A cells, another immortalized, non-tumorigenic MEC cell line, which has been established to execute anoikis in response to loss of anchorage.^11, 12^ In this cell line, the apoptosis program is tightly coupled with growth factor signaling; EGFR expression is downregulated upon loss of adhesion, resulting in upregulation of the Bcl-2 protein Bim.^12^ If this is the case, then it is hypothesized that EGFR expression and signaling is sustained under conditions of detachment in ^Tert^HMECs so that apoptosis is not executed. However, this is unlikely because downregulation of EGFR expression in response to detachment has also been observed in ^Tert^HMECs similar to that in MCF-10A cells (unpublished data). Therefore, the difference between the two cell lines is possibly attributed to the difference in their cell subtypes,^14, 15, 16^ basal/myoepithelial- or luminal-like, although details remain unclear.

### Non-apoptotic cell death in detached ^Tert^HMECs

The identified cell death modalities in detached ^Tert^HMECs include entosis and necrosis along with the activation of the transdifferentiation program resulting in cornification. Given that the majority of malignant breast cancers assume luminal traits, this finding is potentially of great significance as it suggests that metastatic mammary tumors, if not all, acquire resistance to non-apoptotic cell death mechanisms rather than to classical apoptosis and that non-apoptotic cell death mechanisms have the potential to function as an important barrier to the development of breast carcinoma. The tumorigenic role of ERBB2 is also open to alternate interpretations. Although anoikis in MCF-10A cells was suppressed by the activation of ERBB2,^12^ non-apoptotic cell death mechanisms in detached ^Tert^HMECs were apparently unaffected as mentioned above (unpublished data). ERBB2 signaling potentially contributes to tumorigenesis through the regulation of cellular metabolism rather than of cell death.^32^

A critical role of FAK, which engages atypical survival signaling that is tightly coupled with TRAIL expression, emerged in the regulation of non-apoptotic cell death. In contrast to myrFAK, FAK(397E), which is another constitutively active form of the kinase, was unable to suppress DICD (unpublished data); this observation implied that kinase activity alone is insufficient for protecting cells from DICD, and that subcellular localization of the kinase or its anchoring to the plasma membrane, which was achieved by myristoylation, was critical for the regulation of DICD by FAK. On the other hand, recent studies have revealed highly context- and cell type-dependent aspects of FAK signaling.^23^ The present study revealed a novel role of TRAIL in FAK signaling regulating non-apoptotic cell death mechanisms, particularly in entosis (Figure 5), albeit it has been generally accepted as an apoptosis inducer.^37^ A previous study showed that the Rho-dependent actomyosin-mediated contractile system played a role in entosis.^6^ The results obtained in the present study suggest that TRAIL mediates the adhesion-dependent activation of Rho-actomyosin system, although details are yet to be defined. In conclusion, a schematic representation of the key players and their roles in DICD of ^Tert^HMECs is shown in Figure 6h.

The overall phenotype of detached ^Tert^HMECs resembles that of differentiating keratinocytes, which appears natural given that both skin and mammary glands are derived from the ectoderm of the embryo. More specifically, instead of apoptosis, both the cell types activate non-apoptotic cell death mechanisms along with metabolic inactivation upon anchorage loss (Figure 4a, Supplementary Figures S4a and b).^38, 39, 40, 41^ In differentiating keratinocytes, apoptosis is naturally prevented to ensure completion of the differentiation process resulting in non-apoptotic cell death by cornification.^40, 42, 43^ Likewise, under conditions where apoptosis is inhibited, MECs undergo non-apoptotic cell death.^8, 11, 34^ The same regulatory mechanisms that counterbalance apoptosis and non-apoptotic cell death are likely shared between keratinocytes and MECs.

Metastasis is a formidable challenge not only for cancer patients to survive but also for cancer cells to achieve. Millions of cells are possibly released from a primary tumor every day, but only a small minority colonizes a distant organ. Thus, the process of metastasis could be exploited as an opportunity for eradicating cancer cells, particularly during their dormant period, which is probably underpinned by resistance to DICD or anchorage-independent cell growth and survival. This study is expected to set the stage for the identification of relevant molecular targets for interfering with anchorage-independent growth, thereby inducing death in metastasizing breast cancer cells.

## Materials and Methods

### Cell culture and chemicals

HMECs were purchased from Lonza (Walkersville, MD, USA) and cultured in MCDB170 (US Biological, Salem, MA, USA) supplemented with 0.1 mM ethanolamine (Sigma-Aldrich, St Louis, MO, USA), 0.1 mM ortho-phosphoethanolamine (Sigma), 0.25% bovine pituitary extract (Hammond Cell Tech, Windsor, CA, USA), 5 ng/ml EGF (Peprotech, Rocky Hill, NJ, USA), 0.5 *μ*g/ml hydrocortisone (Sigma), 5 *μ*g/ml insulin (Sigma), 5 *μ*g/ml transferrin (Sigma), and 5 *μ*M isoproterenol (Sigma).^44^ For immortalization, HMECs were transduced with hTERT-expressing retrovirus as described previously.^17^

For suspension culture, cells were detached by treatment with 0.025% trypsin, followed by resuspension in the growth medium supplemented with 0.5% methylcellulose and plated on polyHEMA-coated dishes. Anisomycin, pepstatin A, and polyHEMA were obtained from Sigma-Aldrich. E64d and Z-VAD-fmk (Z-VAD) were purchased from Peptide Institute, Inc. (Osaka, Japan), staurosporine (STS) and bafilomycin from Wako Pure Chemical Industries, Ltd. (Osaka, Japan), and necrostatin-1 from Enzo Life Sciences, Inc. (Farmingdale, NY, USA). BD Matrigel Matrix Growth Factor Reduced (GFR) was obtained from BD Biosciences (Franklin Lakes, NJ, USA).

### Expression vectors

The cDNAs for human FAK, catalytic subunit (p110) of PI3K, Bcl-xL, AKT1, AKT2, AKT3, and MEK2 were amplified from HMEC cDNA library and cloned into CSII-CMV-MCS-IRES2-Bsd vector^45^ (CSII vector) with FLAG or HA tag sequences. cDNA for Src was amplified from pcDNA3 chick c-Src^46^ (a generous gift from Dr T Akagi, Osaka Bioscience Institute, Osaka, Japan) and cloned into the CSII vector. The constitutively active Src(Y527F) and MEK2DD were generated using PrimeSTAR Mutagenesis Basal Kit (Takara Bio, Inc., Otsu, Japan). Constitutively active forms of FAK, PI3K, and AKT isoforms were generated by amplification of respective cDNAs using 5′ primers that included c-Src myristoylation signal sequences, followed by cloning into CSII vector. The lentiviral destination vector, CS-RfA-EP, was generated by replacing the gene encoding blasticidin S deaminase in CS-RfA-EB with the gene for puromycin N-acetyltransferase from pGL4.2 vector (Promega, Madison, WI, USA).

For construction of shRNA expression vectors, target sequences for control (SHC002), TRAIL (# 1: TRCN0000005924; # 2: TRCN0000005925), DR4 (TRCN0000005934), DR5 (TRCN0000005933), and p38 (# 1: TRCN0000000510; # 2: TRCN0000000511) shRNAs were obtained from Mission shRNA (Sigma-Aldrich). Oligonucleotides encoding the target sequences were subcloned into pENTR4-H1, and the shRNA expression cassettes were recombined into CS-RfA-EB (for TRAIL, DR4, and DR5) or CS-RfA-EP (for p38) using LR Clonase II (Invitrogen, Carlsbad, CA, USA).

### Infection

Lentivirus production and infection have been previously described.^47^ The selection and maintenance of infected cells was accomplished using 10 *μ*g/ml blasticidin for CSII and CS-RfA-EB vectors and using 1 *μ*g/ml puromycin for CS-RfA-EP constructs.

### Flow cytometry

Cells were washed with PBS/EDTA, digested with 0.25% trypsin, and centrifuged (800 × *g*, 5 min, 4 °C). Following washes with PBS containing 0.2% serum, the cells were resuspended in incubation buffer (10 mM HEPES (pH 7.4), 140 mM NaCl, 5 mM CaCl_2_) containing Annexin V (Annexin-V-FLUOS; Roche Applied Science, Penzberg, Upper Bavaria, Germany) and 1 *μ*g/ml PI (Sigma-Aldrich), incubated for 30 min at room temperature under dark conditions, filtered using 0.4-*μ*m filter, and analyzed using Epics XL (Beckman Coulter, Inc., Fullerton, CA, USA).

### Immunoblot analysis

Immunoblot analysis was conducted as previously described.^45^ The primary antibodies employed in the study are listed in Supplementary Information.

### DNA fragmentation and caspase 3/7 activity assays

DNA fragmentation and activities of caspase 3/7 were assessed using DNA Fragmentation Cell Death ELISA PLUS Kit (Roche Applied Science) and Caspase-Glo 3/7 assay kit (Promega), respectively, according to the manufacturers' instructions.

### Transmission electron microscopy

Cell pellet was fixed with 2.5% (v/v) phosphate-buffered glutaraldehyde for 2 h, washed with phosphate buffer, postfixed in 1% (w/v) phosphate-buffered osmium tetroxide for 1 h at 4 °C, and subjected to TEM as previously described.^48^

### Cell internalization assay

Monolayer cultures of cells were stained with 1 *μ*M solutions of CellTracker Green CMFDA or Red CMTPX (Invitrogen) for 30 min in a CO_2_ incubator. Equal volumes of single-cell suspensions of red and green-labeled cells at densities of 1.0 × 10^5^ cells/ml were mixed in growth media containing 0.5% methylcellulose, and placed on polyHEMA-coated plates. Aliquots of suspended cells were withdrawn at regular intervals and stained with Hoechst 33342 (10 *μ*M; Sigma-Aldrich) for 15 min at room temperature, washed with PBS, and fixed with 3.7% formaldehyde for 10 min at room temperature. Fixed cells were washed with PBS and placed in glass bottom dishes.

Laser scanning confocal microscopy was performed using FV10i-LIV confocal microscope (Olympus Corporation, Shinjuku, Tokyo, Japan). Three-dimensional images were acquired through z-stacking of sequential optical x-y sections taken at 0.5–1.0 *μ*m z-intervals. Orthogonal slice views from z-stack images were processed with the FLUOView software (Olympus).

### TUNEL assay

Cells were fixed with freshly prepared 2% paraformaldehyde for 60 min at room temperature, washed with PBS, and permeabilized with 0.2% Triton X-100 for 2 min at 4 °C. The cells were then incubated with TUNEL reaction mixture containing TUNEL Enzyme and TUNEL label mix (Roche) according to the manufacturer's instructions. Positive staining in the nucleus was identified using FV10i-LIV confocal microscope (Olympus).

### RNA extraction and quantitative RT-PCR

RNA extraction and quantitative RT-PCR were performed as previously described,^47^ with minor modifications. The cDNA samples were mixed with specific primers (Supplementary Information) and GoTaq qPCR Master Mix (Promega), and amplified using MyiQ Real-time PCR detection system (Bio-Rad Laboratories Inc., Hercules, CA, USA) according to the manufacturer's instructions. The mRNA levels were normalized with respect to the control, glyceraldehyde-3-phosphate dehydrogenase (GAPDH).

### LDH release assay

LDH activities were measured using LDH-Cytotoxic Test *Wako* (Wako Pure Chemical Industries, Ltd.). In brief, cells were cultured in polyHEMA-coated 12-well plates containing 1 ml of medium with 0.5% methylcellulose, and precipitated by centrifugation (800 × *g*, 5 min, 4 °C). An aliquot of the supernatant (i.e., medium) was diluted with equivalent volume of PBS containing 0.2% Tween-20 for the quantification of LDH in media. The cell pellet was resuspended in growth medium, and an aliquot of the suspension was similarly mixed with equivalent volume of PBS/0.2% Tween-20, followed by overnight incubation at 4 °C to obtain total LDH levels (media and cells). The mixtures were cleared by centrifugation, incubated with coloring solution, and absorbance at 570 nm was obtained using Appliskan (Thermo Electron Corp., Madison, WI, USA). PBS/0.1% Tween-20 was employed for measuring background. LDH release was evaluated as a ratio of LDH activity in media to total LDH activity (media and cells).

### Statistics

Data are expressed as mean±S.D. unless otherwise indicated. Differences between experimental samples were all analyzed by two-tailed Student's *t-*test. *P*-values less than 0.05 and 0.01 are shown as * and **, respectively. A *P*-value of less than 0.05 was considered as statistically significant.

## Abbreviations

DICD: detachment-induced cell death
DR: death receptor
ECM: extracellular matrix
ERK: extracellular signal-regulated kinase
FAK: focal adhesion kinase
GRB2: growth factor receptor-bound protein 2
^Tert^HMEC: human mammary epithelial cell immortalized with hTERT
ILK: integrin-linked kinase
LDH: lactate dehydrogenase
MAPK: mitogen-activated protein kinase
MEC: mammary epithelial cell
PARP: poly(ADP-ribose) polymerase
PI: propidium iodide
PI3K: phosphatidylinositol-4,5-bisphosphate 3-kinase
polyHEMA: poly(2-hydroxyethyl methacrylate)
shRNA: short hairpin RNA
STS: staurosporine
TEM: transmission electron microscopy
TRAIL: tumor necrosis factor (TNF)-related apoptosis-inducing ligand
Z-VAD (Z-VAD-fmk: *N*-benzyloxycarbonyl-Val-Ala-Asp(O-Me) fluoromethylketone
