# Supplementary material for: Loss of anchorage primarily induces non-apoptotic cell death in a human mammary epithelial cell line under atypical focal adhesion kinase signaling
Source: Cell Death Dis. 2015 Jan 22;6(1):e1619–. doi: 10.1038/cddis.2014.583 (PMC4669778; doi:10.1038/cddis.2014.583)
Supplement: Supplementary Information [file cddis2014583x1.doc]

**Supplementary Information**

**Materials and Methods**

**Antibodies** The following primary antibodies were used for immunoblot analyses: anti-FLAG M2, anti-β-actin, anti-p38, and anti-paxillin antibodies purchased from Sigma-Aldrich (St. Louis, MO, USA); anti-AKT, anti-Bcl-xL, anti-Caspase-3, anti-cleaved caspase-3 (Asp175), anti-PARP, anti-phospho-AKT (S473), anti-phospho-ERK1/2 (T202/Y204), anti-phospho-FAK (Y397), anti-phospho FAK (Y925), anti-phospho-JNK (T183/Y185), anti-phospho-p38 MAPK (T180/Y182), anti-phospho-paxillin (Y31), anti-phospho-Src(Y416), and anti-TRAIL antibodies from Cell Signaling Technology (Boston, MA, USA); anti-FAK, anti-JNK1, and anti-Caspase-14 antibodies from Santa Cruz Biotechnology (Santa Cruz, CA, USA); anti-Filaggrin and anti-Keratin10 antibodies from Covance, and anti-phospho FAK (S910), anti-Src, anti-HA, and anti-ERK1/2 antibodies from Invitrogen (Camarillo, CA, USA), Millipore (Merck Millipore, Oxford, UK), Recenttec Inc. (Taipei, Taiwan), and Zymed Laboratory (San Francisco, CA, USA), respectively.

For flow cytometry analysis, following antibodies were used: PE anti-human CD261 (DR4), PE anti-human CD262 (DR5), APC anti-human CD29 (TS2/16) antibodies from BioLegend (San Diego, CA, USA); PE mouse anti-human CD29 (HUTS-21) antibody from BD Biosciences (Franklin Lakes, NJ, USA).

**Primers for quantitative RT-PCR** The following primers were employed for PCR: Keratin 10 (forward, 5′-GCCTTGAAACAATCCCTGGAA-3′; reverse, 5′-GCTGCACACAGTAGCGACCTT-3′), Filaggrin (forward, 5′-GGCACTGAAAGG CAAAAAGG-3′; reverse, 5′-AAACCCGGATTCACCATAATCA-3′), caspase14 (forward, 5′-CGGCGGATGGCAGAAG-3′; reverse, 5′-GGGTTCGTTTTCCTTGCTT TT-3′), TRAIL (forward, 5′-GCTCTGGGCCGCAAAAT-3′; reverse, 5′-AGGAATGA ATGCCCACTCCTT-3′), death receptor 4 or DR4 (forward, 5′-GCAGCTGGAC CTCACGAAAA-3′; reverse, 5′-CCTGGGCCTGCTGTACCA-3′), death receptor 5 or DR5 (forward, 5′-GGCCACAGGGACACCTTGTA-3′; reverse, 5′-TCGCCCGGT TTTGTTGA-3′), and GAPDH (forward, 5′-CCAGGTGGTCTCCTCTGACTTC-3′; reverse, 5′-GTGG TCGTTGAGGGCAATG-3′).

**Determination of ATP/ADP ratio and mitochondrial membrane potential (ΔΨm)** ATP/ADP ratio was measured using EnzyLight ADP/ATP Ratio assay kit (BioAssay Systems, Hayward, CA, USA) according to the manufacturer’s instructions. ΔΨm was determined using Mito-ID® Membrane Potential Cytotoxicity Kit (Enzo Life Sciences) as previously described[^1^](#_ENREF_1).

**Transient transfection and indirect immunocytochemistry.** Cells were transiently transfected with X-tremeGENE 9 (Promega), according to the manufacturer’s instructions. At 24 h after transfection, cells were detached and cultured on cover slips for another 24 h. Cells grown on cover slips were washed with PBS and fixed with methanol for 2 min at room temperature. Fixed cells were blocked with 3% BSA in TBS, containing 0.05% Tween 20 (TBS-T), for 30 min and incubated with anti-FLAG M2 antibody for 1 h. After washing three times with TBS-T for 5 min, cells were incubated with Alexa 568-conjugated secondary antibody (Life Technologies) for 1 h. Cells were washed three times with TBS-T for 5 min and then incubated with 100 ng/ml of 4′, 6-diamidino-2-phenylindole (DAPI) for 2 min. All images were obtained using a FluoView FV10i (OLYMPUS, Tokyo, Japan).

**Protein expression analysis on cell surface and β1 integrin activity assay.** To analyze protein expression on cell surface, cells were treated with TrypLE Express (Life Technologies), washed with FACS buffer (0.5% BSA/PBS), and then stained with antibodies that conjugated to phycoerythrin (PE) or allophycocyanin (APC) for 20 min at 4°C. After washing twice with FACS buffer, cells were analyzed using the MoFlo AstriosEQ (Beckman Coulter, Inc., Fullerton, CA).

The activation of β1 integrin was examined, as described by Gutierrez-Lopez MD et al[^2^](#_ENREF_2). In brief, cells were washed with PBS that included 0.53 mM EDTA, treated with Accumax (Innovative Cell Technologies) for 5 min at 37°C, and collected into a microtube. After washing with modified Tyrode’s buffer (5 mM Hepes at pH 7.4, 137 mM NaCl, 2.7 mM KCl, 1 mM MgCl_2_, 0.5 mM CaCl_2_, 12 mM NaHCO_3_, 0.1% of glucose, 0.1% of BSA), cells were incubated at 37°C for 20 min with a stimulatory anti-human β1 integrin antibody (TS2/16) conjugated to APC in modified Tyrode’s buffer and subsequently mixed with 1 volume of prediluted anti-activated β1 integrin antibody (HUTS-21) conjugated to PE. After an additional incubation at 37°C for 30 min, cells were washed twice with modified Tyrode’s buffer and analyzed using the MoFlo AstriosEQ (Beckman Coulter, Inc., Fullerton, CA). Mean fluorescence intensities were calculated using the Kaluza software (Beckman Coulter, Inc., Fullerton, CA).

**Figure Legends**

**Figure S1** FACS analysis of DICD, and its dependence on the presence of ECM. (**a**) ^Tert^HMECs were cultured as monolayers (A) or in suspension (S) for 48 h, stained with PI and Annexin V, and subjected to flow cytometry, as described in Figure 1**a**. The percentages of subpopulations in the regions of the blot are shown. For experimental control, monolayer cultures were treated with 0.5 µM staurosporine (STS) in the presence or absence of 50 µM Z-VAD-fmk (Z-VAD) for 6 h. (**b**) ^Tert^HMECs were cultured as monolayers (A) or in suspension (S) for 48 h in the presence or absence of 5% of matrigel (Mtx). The percentage of PI-positive subpopulation was measured using flow cytometry, and depicted as a graph, as above.

**Figure S2** DICD regulation by myrFLAG-FAK. (**a**) ^Tert^HMECs, which were transiently transfected with myrFLAG-FAK, were subjected to indirect immunocytochemistry using anti-FLAG antibody. Nuclei were labeled with DAPI. Scale bars: 10 µm. (**b**) Cells that stably expressed myrFLAG-FAK were cultured for 24 h as monolayers (A) or in suspension (S) and examined by immunoblotting using antibodies, as indicated. β-actin was used as a loading control. (**c**) Cells that expressed myrFLAG-FAK were cultured for 48 h, as shown in (**b**), stained with PI and Annexin V, and analyzed by flow cytometry, as shown in Figure S1**a**. The percentages of subpopulations in the regions of the blot are shown. (**d**) Cells that expressed myrFLAG-FAK were cultured as shown in (**c**), stained with anti-activated β1 integrin (HUTS21) and total β1 integrin (TS2/16) antibodies, and were subjected to flow cytometry analysis, as described in Materials and Methods. β1 integrin activity was determined as ratio of mean fluorescent intensity of HUTS-21 to that of TS2/16. The ratios relative to those in monolayer (A) cultures of Mock-infected cells are shown. Values represent the means ± S.D. from at least three independent experiments. **P < 0.01

**Figure S3.** DICD in ^Tert^HMECs is independent of survival signaling regulated by AKT, MEK2, and Bcl-xL. (**a,** **c,** **e**) ^Tert^HMECs expressing myrAKT (myrHA-AKT) (**a**), MEK2DD (**c**), and Bcl-xL (**e**) were cultured as monolayers (A) or in suspension (S) for 24 h, and examined by immunoblot analysis using the indicated antibodies. β-actin was employed as loading control. (**b,** **d**) Cells were cultured for 48 h as in (**a, c**), followed by staining with PI and Annexin V, and analyzed by flow cytometry as shown in Figure S1**a**. The percentage of PI-positive subpopulation was depicted as a graph. (**f**) Cells expressing Bcl-xL were cultured for 24 h in the presence or absence of 1 μM STS, stained with PI and Annexin V, and subjected to flow cytometry analysis as above. The percentages of PI- and Annexin V-positive subpopulations are depicted as a graph. Values represent means ± S.D. from at least three independent experiments. **, p < 0.01

**Figure S4.** DICD in ^Tert^HMECs is accompanied by deterioration of energy metabolism. (**a**) ^Tert^HMECs were cultured for 24 h as in Figure S1**a** and **b**, and ATP/ADP ratio was measured using EnzyLight^TM^ ADP/ATP Ratio Assay Kit. The ATP/ADP ratio was normalized with respect to monolayer (A) cultures, and depicted as graph. Values represent means ± S.D. from at least three independent experiments, with measurements made in triplicate for each experiment. (**b**) Cells were cultured for 24 h as in (**a**), and ΔΨm was measured with Mito-ID® Membrane potential cytotoxicity kit. Measurements were made in triplicate, and ratios were normalized with respect to monolayer cultures and depicted as graph. Data represent means ± S.D. from three independent experiments. **, p < 0.01

**Figure S5.** Potential role of DR4 as a receptor for TRAIL signaling in DICD. (**a**) ^Tert^HMECs were cultured in suspension for the indicated time periods, and TRAIL mRNA levels were analyzed by quantitative RT-PCR, as detailed in Figure 5**a**. (**b**) Cells expressing shRNAs (Ctr: control, DR4, DR5) were cultured as monolayers (A) or in suspension (S) for 24 h, and the levels of DR4 and DR5 mRNA were analyzed by quantitative RT-PCR. (**c**) Cells expressing the shRNAs were cultured for 24 h as in (**b**), stained with PE-labeled antibodies against DR4 or DR5, and analyzed for their mean fluorescent intensities by flow cytometry analysis, as described in Materials and Methods. (**d**) Cells expressing the shRNAs were cultured for 48 h as in (**b**), and subjected to flow cytometry analysis as shown in Figure S1**a**. The percentage of PI-positive subpopulation was depicted as a graph. Data represent means ± S.D. from at least three independent experiments. *, p < 0.05; **, p < 0.01; N.S., not significant

**Figure S6.** The effect of p38 MAPK inhibitor, SB203580, on the expression of cornification markers. ^Tert^HMECs were cultured in the presence or absence of 5 μM SB203580 as monolayers (A) or in suspension (S) for 24 h, and mRNA and protein levels were analyzed as in Figure 4**a**. Values represent means ± S.D. from at least three independent experiments. *, p < 0.05; **, p < 0.01

**References**

1. Ishikawa F, Kaneko E, Sugimoto T, Ishijima T, Wakamatsu M, Yuasa A *et al.* A mitochondrial thioredoxin-sensitive mechanism regulates TGF-beta-mediated gene expression associated with epithelial-mesenchymal transition. *Biochemical and biophysical research communications* 2014; **443**(3)**:** 821-7.

2. Gutierrez-Lopez MD, Ovalle S, Yanez-Mo M, Sanchez-Sanchez N, Rubinstein E, Olmo N *et al.* A functionally relevant conformational epitope on the CD9 tetraspanin depends on the association with activated beta1 integrin. *The Journal of biological chemistry* 2003; **278**(1)**:** 208-18.
