# Supplementary figures and images for: Loss of anchorage primarily induces non-apoptotic cell death in a human mammary epithelial cell line under atypical focal adhesion kinase signaling
Source: Cell Death Dis. 2015 Jan 22;6(1):e1619–. doi: 10.1038/cddis.2014.583 (PMC4669778; doi:10.1038/cddis.2014.583)

a

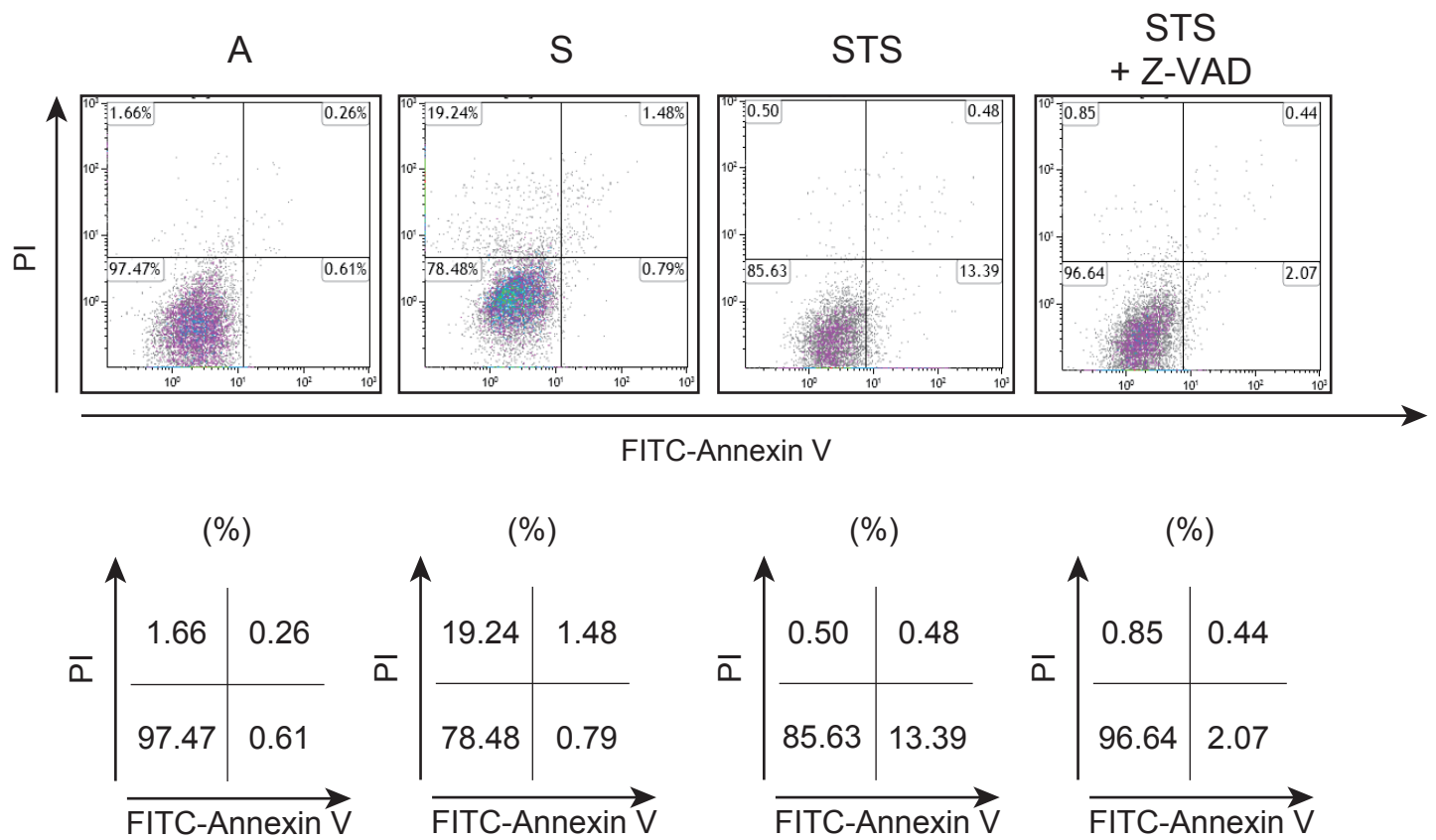

b

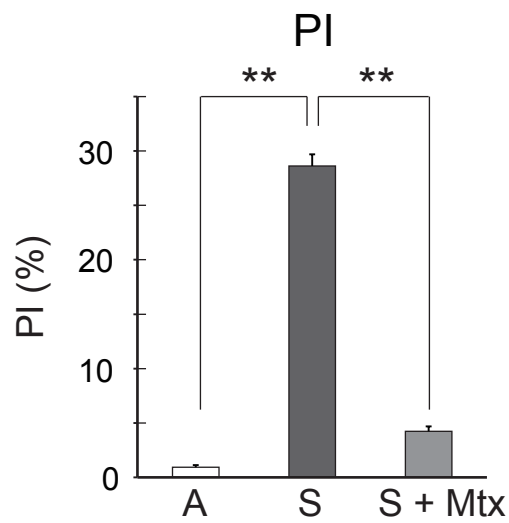

Supplement: Supplementary Figure S1 [file cddis2014583x2.pdf]

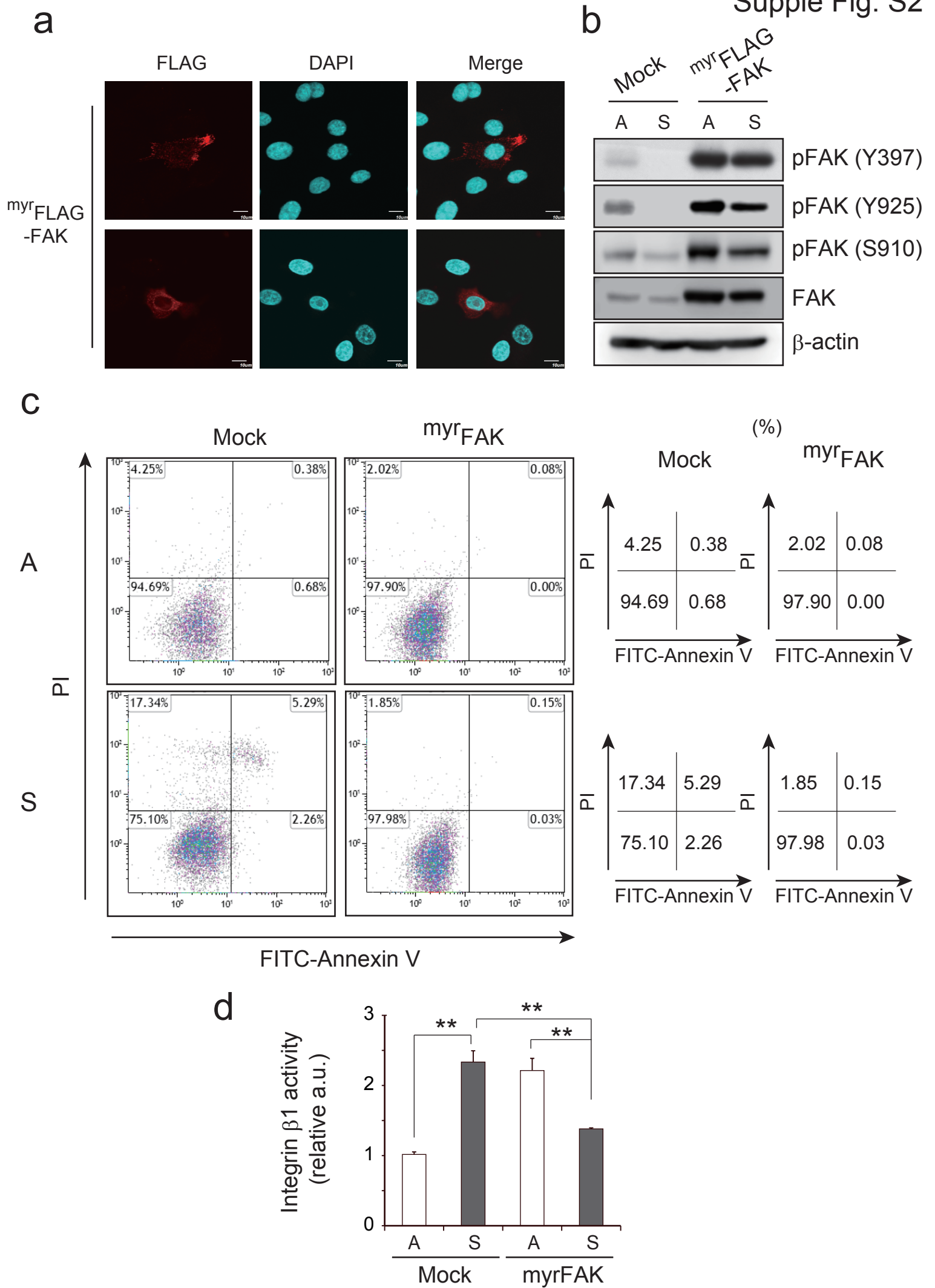

Supplement: Supplementary Figure S2 [file cddis2014583x3.pdf]

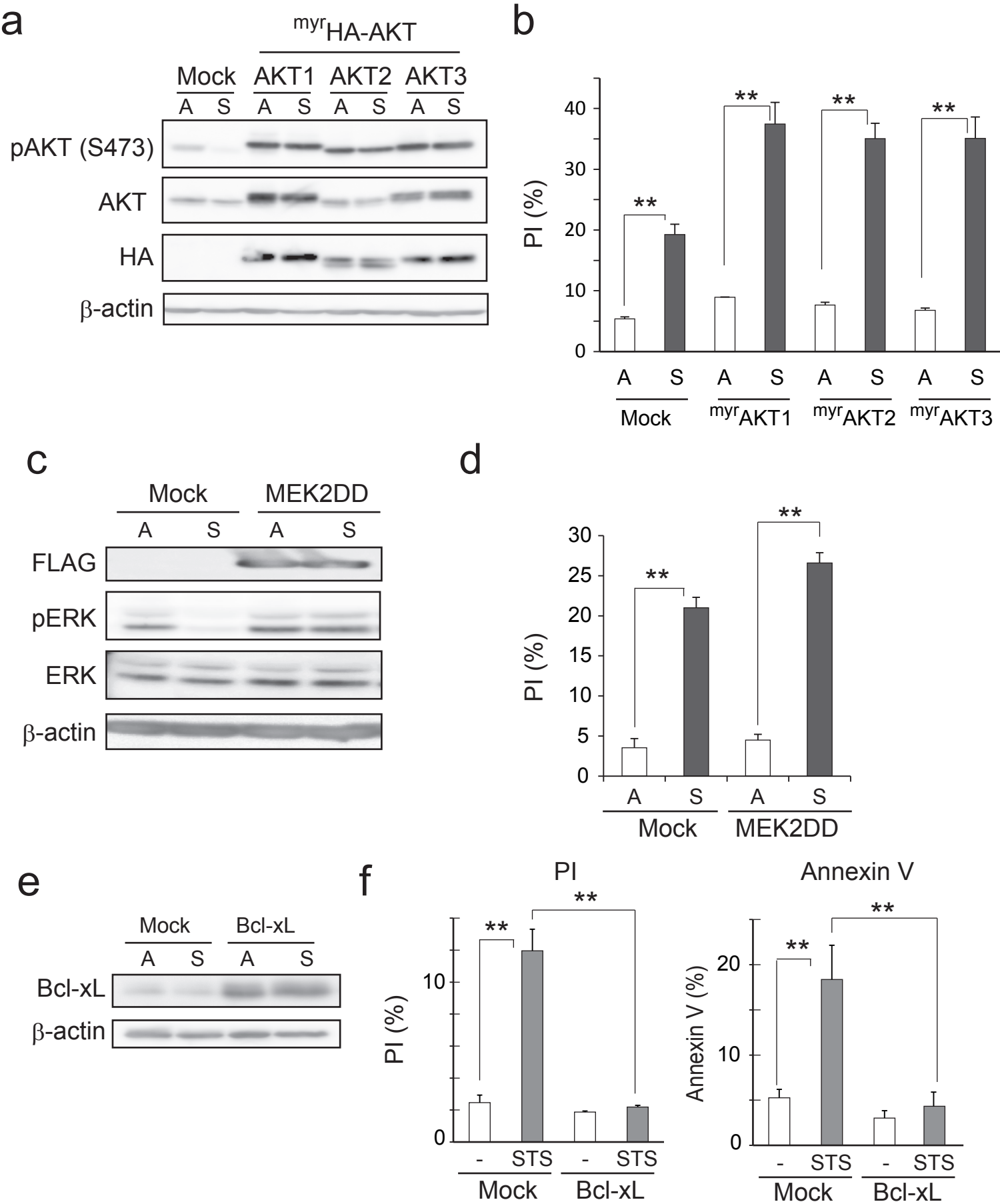

Supplement: Supplementary Figure S3 [file cddis2014583x4.pdf]

a

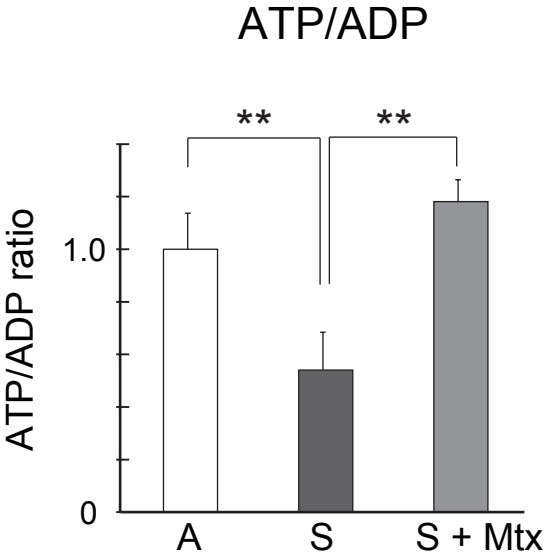

b

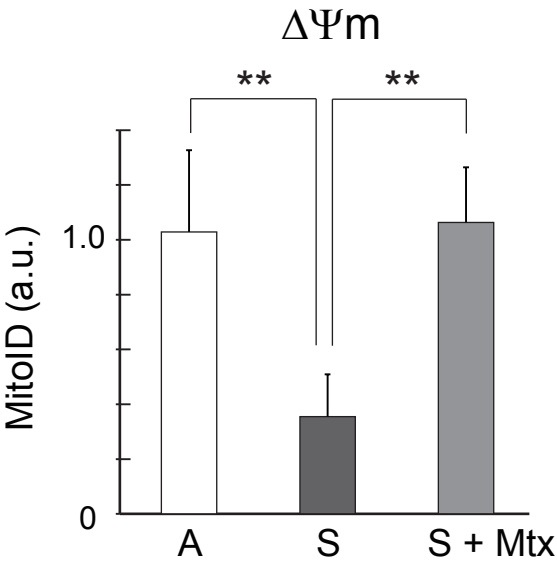

Supplement: Supplementary Figure S4 [file cddis2014583x5.pdf]

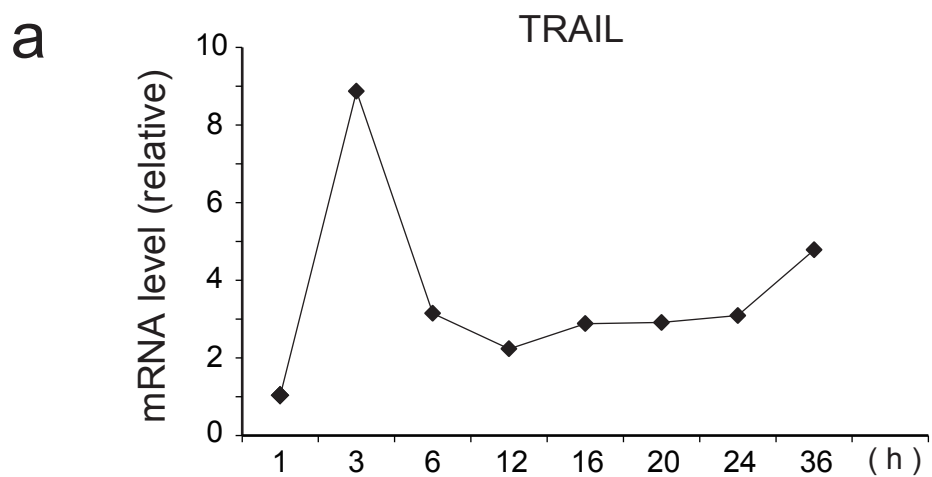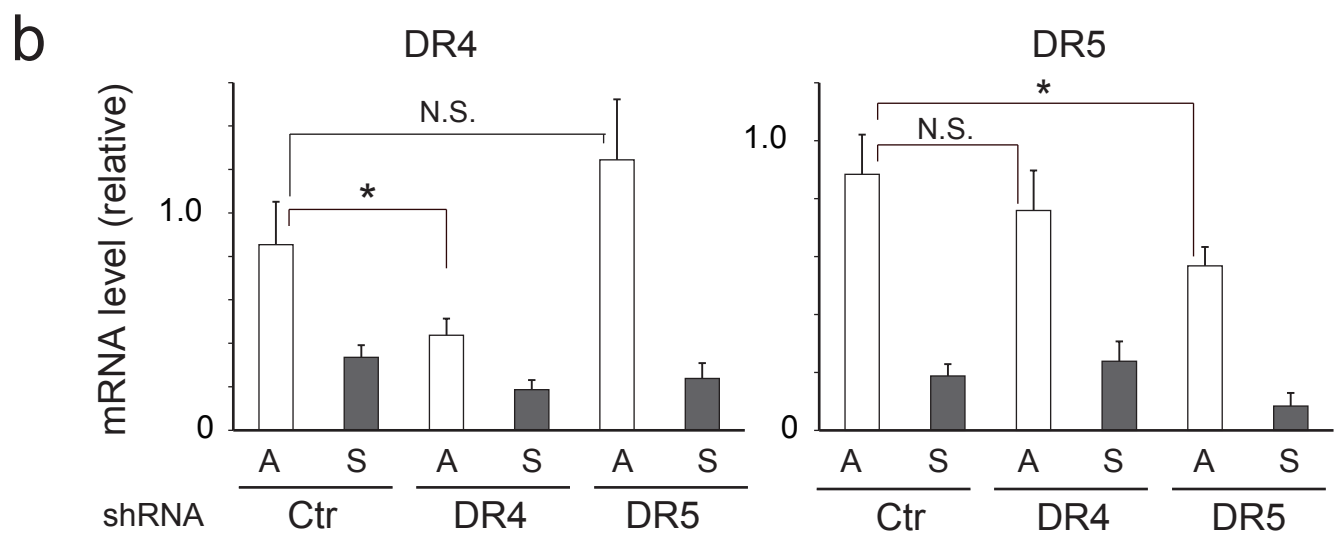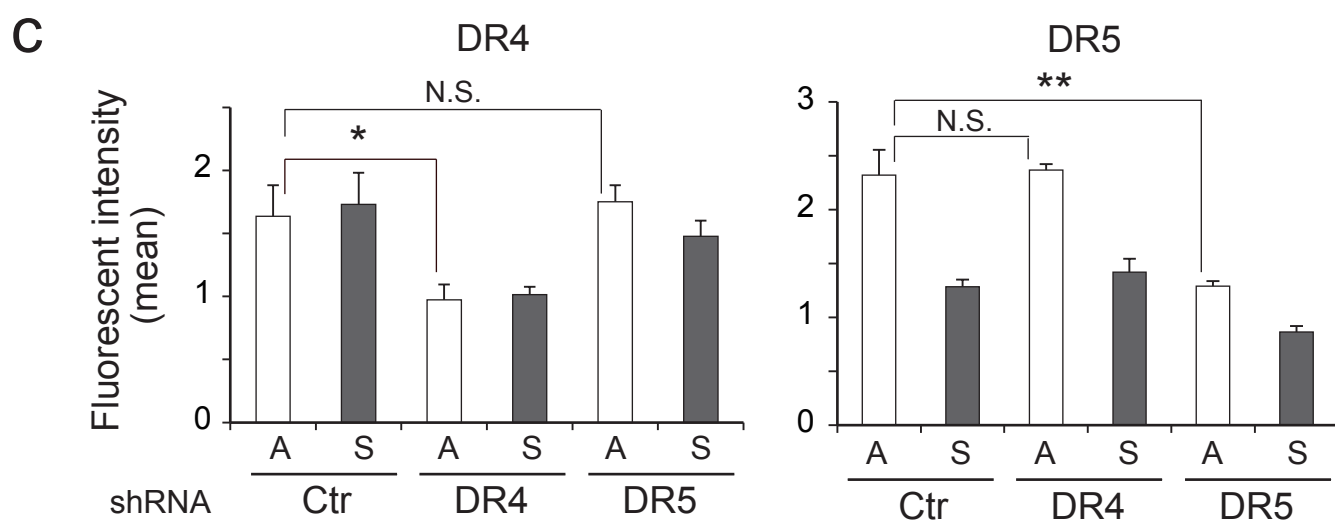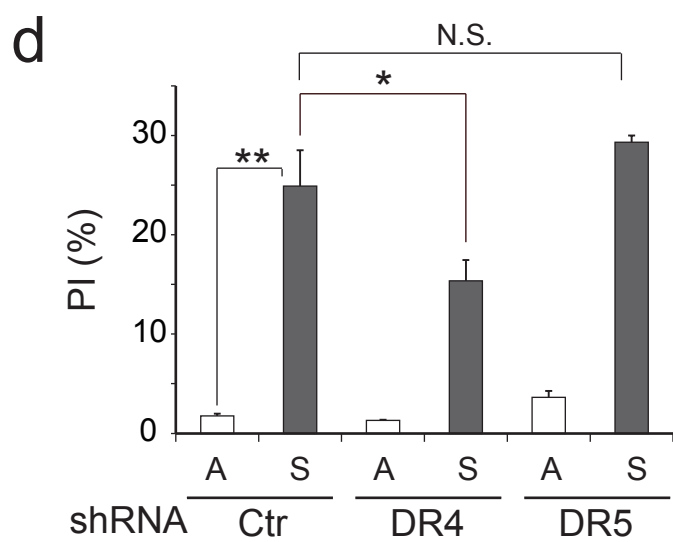

Supplement: Supplementary Figure S5 [file cddis2014583x6.pdf]

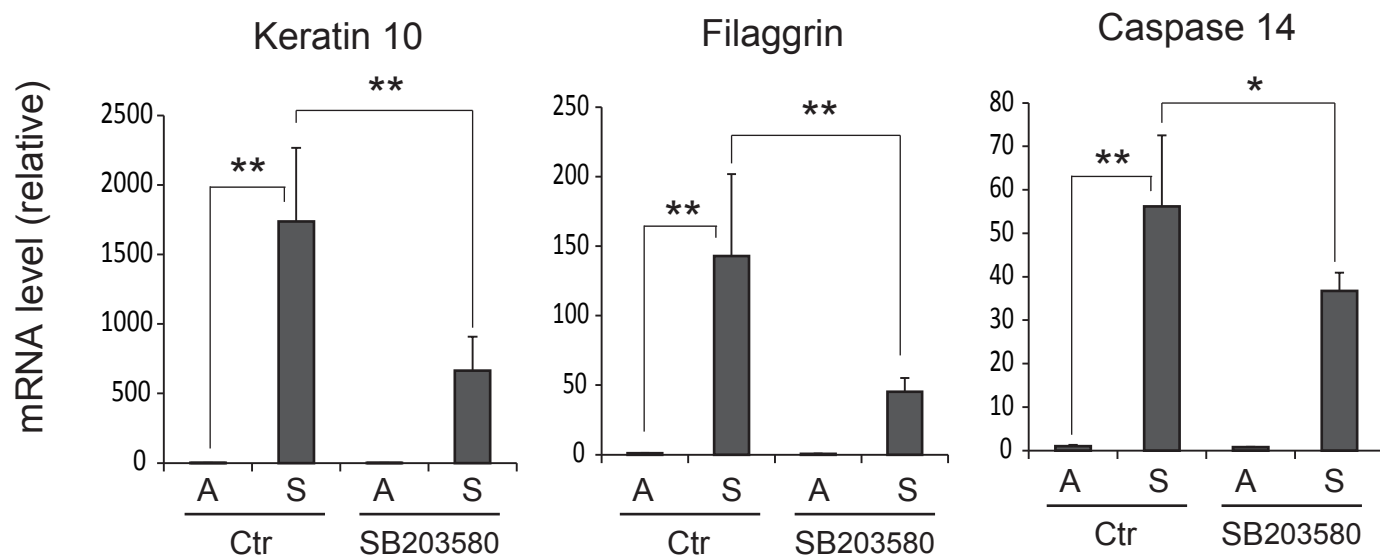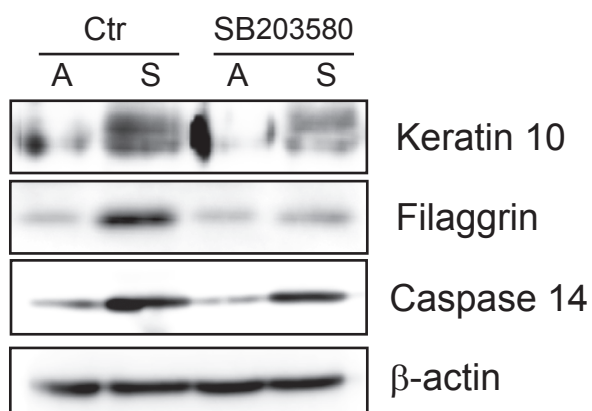

Supplement: Supplementary Figure S6 [file cddis2014583x7.pdf]
